# Can Machines Think? Interaction and Perspective Taking with Robots Investigated via fMRI

**DOI:** 10.1371/journal.pone.0002597

**Published:** 2008-07-09

**Authors:** Sören Krach, Frank Hegel, Britta Wrede, Gerhard Sagerer, Ferdinand Binkofski, Tilo Kircher

**Affiliations:** 1 Department of Psychiatry, RWTH Aachen University, Aachen, Germany; 2 Central Service Facility “Functional Imaging” at the ICCR-BIOMAT, RWTH Aachen University, Aachen, Germany; 3 Technical Faculty, Cognitive Interaction Technology, Bielefeld University, Bielefeld, Germany; 4 Department of Neurology, University Hospital Schleswig-Holstein, Lübeck, Germany; 5 NeuroImage Nord, Department of Systemic Neuroscience, University Medical Center, Hamburg, Germany; 6 Brain Imaging Centre West (BICW), Forschungszentrum Jülich, Wilhelm-Johnen Straße, Jülich, Germany; Harvard Medical School, United States of America

## Abstract

**Background:**

When our PC goes on strike again we tend to curse it as if it were a human being. Why and under which circumstances do we attribute human-like properties to machines? Although humans increasingly interact directly with machines it remains unclear whether humans implicitly attribute intentions to them and, if so, whether such interactions resemble human-human interactions on a neural level. In social cognitive neuroscience the ability to attribute intentions and desires to others is being referred to as having a Theory of Mind (ToM). With the present study we investigated whether an increase of human-likeness of interaction partners modulates the participants' ToM associated cortical activity.

**Methodology/Principal Findings:**

By means of functional magnetic resonance imaging (subjects n = 20) we investigated cortical activity modulation during highly interactive human-robot game. Increasing degrees of human-likeness for the game partner were introduced by means of a computer partner, a functional robot, an anthropomorphic robot and a human partner. The classical iterated prisoner's dilemma game was applied as experimental task which allowed for an implicit detection of ToM associated cortical activity. During the experiment participants always played against a random sequence unknowingly to them. Irrespective of the surmised interaction partners' responses participants indicated having experienced more fun and competition in the interaction with increasing human-like features of their partners. Parametric modulation of the functional imaging data revealed a highly significant linear increase of cortical activity in the medial frontal cortex as well as in the right temporo-parietal junction in correspondence with the increase of human-likeness of the interaction partner (computer<functional robot<anthropomorphic robot<human).

**Conclusions/Significance:**

Both regions correlating with the degree of human-likeness, the medial frontal cortex and the right temporo-parietal junction, have been associated with Theory-of-Mind. The results demonstrate that the tendency to build a model of another's mind linearly increases with its perceived human-likeness. Moreover, the present data provides first evidence of a contribution of higher human cognitive functions such as ToM in direct interactions with artificial robots. Our results shed light on the long-lasting psychological and philosophical debate regarding human-machine interaction and the question of what makes humans being perceived as human.

## Introduction

“Can machines think?”- This pivotal question was raised by Alan Turing in 1950 however, philosophers debate this issue since the early 17^th^ century [1]. In the present study we address the related question of whether humans implicitly think that machines can think. More exactly, what would happen if machines behaved and appeared human-like? Would, in such a scenario, humans ascribe futuristic machines, as e.g. social, humanoid robots mental qualities [2]? Would humans assign intention, volition and rational choices to such humanoid robots and would attributions vary with the perceived grade of human-likeness of the machine? In the first place, von Kempelen approached these questions by introducing the so-called “chess-turk” in 1769. The chess-turk incorporated a life-size figure sitting in front of a chess-board while moving pieces with its own hands. Spectators and opponents, however, were not aware of that pieces were moved by a real human hidden inside the box. Hence, the chess-turk was the first extensively documented example of attributed intelligence to an anthropomorphic agent [3].

Besides principle theoretical interests in mental state attribution, applied cognitive neurosciences have increasingly addressed this issue. Inferring intentions, goals or desires of others is highly advantageous for successful interpersonal communication. Further, understanding and foreseeing the mental states of others and thus, building a model of others' minds, enables us to empathize but also to manipulate the behaviour of conspecifics. In psychology the ability to build a model of another's mind has been referred to as mentalizing or having a Theory of Mind (ToM) [4]. According to Saxe [5], humans intuitively conceive humans as having a mind and attribute them contentful mental states, but does that account for agents dissimilating human beings, as e.g. humanoid robots, too?

In general, mentalizing requires the ability to differentiate animate and inanimate entities [6], to share attention by following the gaze of another agent [7], to represent goal-directed actions [8] and to distinguish between actions of the self and of others [9], [10]. Sources of information about the inner states of another person are behavioural patterns, such as eye gaze direction, body and facial movements, which, in conjunction with the representation of our own mental states, allow to infer the intentions of that agent [11], [12]. In recent years, neural correlates underlying mental state attribution have been extensively investigated using a number of different approaches: Studies investigating ToM by means of functional magnetic resonance imaging (fMRI) usually ask participants to take the perspective of various stimuli types e.g. cartoon characters [13]–[15], persons on a photograph [16], or even geometrical shapes chasing each other [17]. However, for these kinds of tasks, subjects are asked to evaluate ToM situations from an explicit, and therefore highly controlled perspective.

In contrast, more recent neuroimaging studies focused on an implicit detection of ToM by applying reciprocal interactive games [18]–[24]. Here, tasks employed include variations of the iterated Prisoner's Dilemma Game (PDG), and others such as the ultimatum game [19], stone-paper-scissor game [22], a coloured disc pattern game [21] or economic decision games as e.g. Iowa Gambling Task [23].

However, until now functional neuroimaging studies have only concentrated on human game partners in comparison to computer partners. Recent findings indicate that humans do attribute self-generated actions, intentions and desires rather to human than to computer partners, though activity in the mentalizing network was detected in the human-computer interaction as well, especially when the computer was perceived being directly responsive to the human partner's behavioural decisions [19], [22].

The neural network associated with mentalizing comprises at least two circumscribed brain regions: the right posterior superior temporal sulcus (pSTS) at the temporo-parietal junction (TPJ) and the medial prefrontal cortex (mPFC) [12], [14], [25]–[29]. According to Decety and colleagues the TPJ thereafter depicts a heteromodal association cortex integrating input from lateral and posterior thalamus while in turn holding reciprocal connections to the prefrontal cortex and to the temporal lobes [30]. The TPJ is considered as being sensitive to naturalistic biological motion and human eye gaze observation [31], hence, affine to stimuli signalling intentional activity [13]. Further, the distinction between self-produced actions and actions generated by others, termed agency, is linked with the TPJ and its vicinity [32]–[35]. The medial prefrontal cortex (medPFC) and the adjacent paracingulate cortex are activated when participants switch into the “view of the world” of another person and anticipate what a person is going to think, feel or do by considering what he or she would think, feel or do being in the same situation [12], [36].

**Figure 1:**
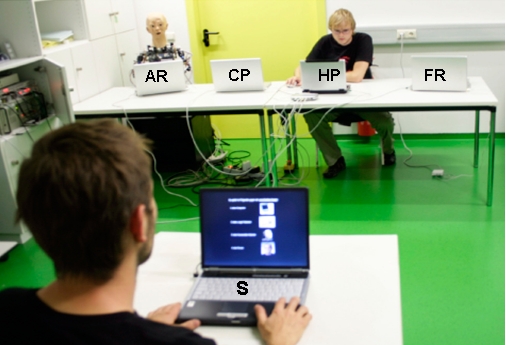
Arrangement of the briefing; from left to right in the back: anthropomorphic robot (AR), computer partner (CP), human partner (HP) and functional robot (FR); in front: subject (S).

Referring to the above mentioned research question of whether humans might attribute intention or agency to non-human interactors and especially to non-human interactors that behave and appear human-like, we engaged participants in a highly interactive game scenario with four opponents, all hypothetically differing linearly in the perceived grade of human-likeness (see Fig. 1): a computer partner, a functionally designed LEGO mindstorm robot, an anthropomorphic robot (BARTHOC Jr.) and a human partner.

Human-likeness was operationalized by increasing degrees of embodiment and anthropomorphism of game partners: embodiment refers to the perception of a physical interaction as a requirement to attribute intelligence to someone or something, while anthropomorphism is described as the tendency to attribute human characteristics to objects and animals in order to interpret their actions in an understandable way. According to v. Foerster humans anthropomorphize because that allows us to explain things we do not understand in terms that we do understand, and what we understand best is ourselves [37]. Consequently, Duffy argues that robots need to have a certain degree of anthropomorphic attributes in order to allow for a meaningful social interaction with a human being [38]. DiSalvo and colleagues speculate that the degree to which people anthropomorphize depends on the quantity of human attributes [39]. Therefore, the more human attributes an artificial agent displays the more it is rated as human-like and accordingly more people attribute human qualities to the nonhuman agent [40].

The embodiment of a robot may also have an effect on the human-likeness due to the feeling of social presence when interacting with a robot. The feeling of social presence will be enhanced by physical embodiment of an agent [41]. According to Dautenhahn et al. robots have different relationships with the world depending on their sensorimotor capabilities. Some robots are more embodied than others because of their degree of perturbatory bandwidth, i.e. the mutual perturbations between an agent and its environment [42]. Therefore, the anthropomorphic robot BARTHOC Jr. is able to perturb the social environment to a greater extent than the functionally designed LEGO mindstorm robot or the computer.

Regarding anthropomorphism, Epley and colleagues postulate three factors that determine the extent to which people anthropomorphize: (a) Elicited Agent Knowledge: Knowledge about humans in general or self-knowledge serve as a basis for induction primarily because such knowledge is acquired earlier and is more richly detailed than knowledge about nonhuman agents or objects. The more similar in appearance or motion the nonhuman agent is the more people are likely to use themselves as a source of induction. For example, robots are anthropomorphized more readily when given human-like faces or bodies and hummingbirds suddenly appear more deliberate and thoughtful when their natural quickness is slowed to a human-like speed [43]. (b) Effectance Motivation: Effectance describes the need to interact effectively with one's environment. Attributing human characteristics and motivations to nonhuman agents increases the ability to make sense of an agent's action and reduces uncertainty. Finally, (c) Sociality Motivation describes the need and desire to establish social connections with other humans. When people feel lack of social connection they anthropomorphize to a higher content to satisfy their motivation to be together with others. The physiognomy of a robot changes the perception of its human-likeness, knowledge, and sociability. Therefore, people avoid negatively behaving or looking robots and prefer to interact with positive robots [44]. Furthermore, an expressive face indicating attention [45] and imitating the face of a user [46] makes a robot more compelling to interact with.

Especially as machines become fixtures at home and workplace, our interactions with them will become more sophisticated and inevitable. Within this context it has been proposed that social robots serve as an interface between humans and technology with the supposition that the more anthropomorphic a robot looks like the more the user will expect the robot to behave like a human being [47]. We assume that a human-like looking and behaving robot is the easiest to use interface simply because humans are highly skilled in having natural interaction with other humans. As the form and appearance of robots has substantial influences on the assumptions humans have about specific applications and behaviours [48], four categories of robotic forms can be differentiated: anthropomorphic, zoomorphic, caricatured, and functional [49], with the functional form being farthest away from human-likeness. Zoomorphic appearing robots are intended to look like their animal counterparts to support the idea that an observer expects the robot to behave like an animal. In some cases this might be helpful to communicate the functional limitations of a robot. For example, a dog is partly able to understand aspects of human speech, but makes many mistakes. This mirrors the quality of speech recognition software. Robots with a caricatured appearance, however, are mainly designed both to not elicit any familiar expectations and to focus on very specific attributes like mouth or eyes.

Here we decided to fill the gap between the assumingly low end of attributed mental states-a computer partner-and the obviously high end-a real human partner-with the two robotic forms, functional and anthropomorphic, postulated by Fong et al [49].

**Figure 2:**
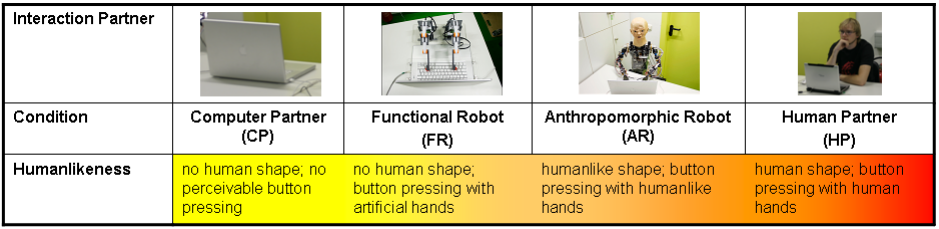
Surmised game partners (abbreviations) with increasing degrees in human-likeness.

The present study focuses on two major questions: Firstly, do humans attribute intentions and self-initiated, rational decisions to robotic agents and secondly, do differing grades of human-likeness of interactors mediate such mental state attributions? Therefore we applied a version of the classical iterated PDG with subjects instructed to play four mocked confederates. We assume that, irrespective of the response behaviour of the interactors, which unknowingly to the participants was randomized in advance, participants would increasingly express fun playing the game, engage in the interaction and suffer from loosing the more interactors displayed human-like features (CP<FR<AR<HP; see Fig. 2). Further, activity of the mentalizing network, i.e. the right temporo-parietal junction (TPJ) and the medial prefrontal cortex (medPFC), was hypothesized to linearly increase with the perceived grade of human-likeness of the interactors.

## Materials and Methods

Twenty consenting healthy male subjects with an average age of 24.5 years (SD = 2.97) took part in the study and were paid a fee for participation. In order to control for possible cognitive factors that may influence the performance on the task neuropsychological testing comprising attention [50], executive functions [51] and IQ [52] was administered. Participants were excluded if they had been diagnosed with a past or present psychiatric, neurological, or medical disease as well as with psycho-pharmacological medication intake at time of study or within the last two months. According to the declaration of Helsinki the study was approved by the local ethics committee.

### Game scenario

Prior to scanning, participants were familiarized with the game scenario and the mocked game partners of the present study. The game resembled the scenario described in the classical iterated prisoners' dilemma (PDG) [53]. In this game two players are faced with the same decision: cooperate with each other or defect. Both players may gain a previously defined sum of points depending on both, their own as well as their counterpart's decision, i.e. left or right button pressing. The dilemma eventuates such that a unilateral (selfish) win of one player is maximised by defection, whilst the counterpart cooperates, but punished bilaterally if both players defect. Hence, relying on mutual cooperation – yielding high shared earnings – comes along with the risk of being deceived. The iterated PDG evokes a taking over of another's perspective and thereby allows to implicitly tap into ToM processes. Depending on the interactor's decision, the participant immediately received the previously defined and explicitly learned pay-off feedback, making the scenario highly interactive. In the present study both contenders (participant and either CP, FR, AR or HP; see Fig. 1) would be gratified with 20 points when simultaneously pressing the left button (CC). Pressing the left button (cooperation) with the respective partner pressing the right button at the same time (defection), the further would return with 10 points for this game, while the latter receives 20 points (CD) and vice versa (DC). In case both contenders choose to defect, the dilemma eventuates with both sides receiving zero points (DD). CC implies mutual cooperation, while DD involves mutual non-cooperation [19].

The decision matrix resembled matrices already applied by other research groups [19], [20], [24] and was validated in a pre-test to meet our criteria. Interactions with game partners were interspersed by a low-level baseline condition enforcing participants to alternately press the right and left button when a central crosshair appeared on the computer screen. Importantly, the instruction given to the participants involved the demand to both, “win a series of games and reach a virtual highscore”. As these two converse goals could, per definition, not be reached by solely pressing one button, this matrix secured an almost equal pressing of both buttons, thereby supporting the idea behind: finding a decision based upon the reasoning about the opponent's last decisions (‘I think that you think that I think…’), i.e. triggering Theory of Mind processes.

### Setting of the briefing

After introducing participants to their game partners (CP, FR, AR and HP) for the upcoming game sessions, participants were seated face-to-face with their interactors (see Fig. 1). All participators had a commercial notebook located at their side of the table. Via the notebook, which was placed in front of the participant, the instruction of the experiment was displayed. A mocked connecting cable linked the participant's notebook with all four notebooks of putative game partners.

For the briefing both robots were programmed in advance to push their keyboard buttons exactly at the same time when the participant believed to play them. Similarly, the confederate (always the same male research associate S.G.) simultaneously pressed his buttons when the subject assumed playing the human partner. During the computer condition no movement was noticeable. Furthermore, at the time of pausing, the anthropomorphic robot was programmed to orient towards the momentarily playing “colleague” or the participant (Supporting Information Video S1). However, during the tutorial as well as during the entire experiment, the response behavior of all four game partners (CP, FR, AR and HP) was randomized unknowingly to the participant. By this means a real cooperation or search of “a best way” was avoided.

**Figure 3:**
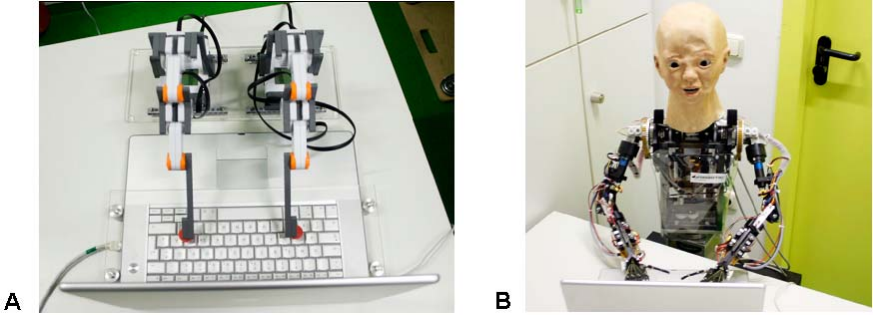
A: Functional Robot, Lego Mindstorms; custom-made keyboard button system; B: Anthropomorphic Robot, BARTHOC Jr. (Bielefeld Anthropomorphic RoboT for Human-Oriented Communication).

According to theories on embodiment the functional robot was designed to solely press buttons, while other human-like features were totally omitted (see Fig. 3). The anthropomorphic robot BARTHOC Jr. (Bielefeld Anthropomorphic RoboT for Human-Oriented Communication) likewise fulfilled the criteria of embodiment, thus furthermore displayed increased human-likeness due to his anthropomorphic design:

#### a) The Functional Robot (FR)

The functional robot with its two arms (see Fig. 3) was constructed of two Lego Mindstorm sets (http://mindstorms.lego.com). Each arm consists of two servomotors and a Lego NXT controller (i.e. a computer controlled Lego brick). The two servomotors are directly connected to the NXT controller. The movements of the servomotors are very precise (+/−1°) so that a believable button pressing animation on the computer keyboard is warranted. The button pressing behavior was programmed with the Mindstorms NXT software that serves as an intuitive drag and drop programming software to design robots. The functional design represents two arms modeled after a human arm to support the idea of low human-likeness.

#### b) The Anthropomorphic Robot BARTHOC Jr

BARTHOC Jr. looks like a child at the age of five years with the size of 65 cm from the waist upward (see Fig. 3). The robot is able to move its torso which is mounted on a 65 cm high chair like socket to the left and to the right. The socket includes the power supply, actuator controllers (so-called iModules), and two serial interfaces to a computer. One interface controls the head and neck actuators, the other one is connected to the actuators below the neck. In total 41 actuators consisting of DC- and servomotors navigate the robot. The face has ten degrees of freedom to control jaw, mouth angles, eyes, eyebrows, and eyelids. The eyes are vertically aligned and horizontally moveable. Furthermore, the head can be turned, tilted to its side and slightly shifted forwards and backwards. Each arm can be moved similar to the movements of a human arm. With its five fingers on each hand BARTHOC Jr. is able to show simple grips as well as deictic gestures.

However, note that the assumptions about the degree of human-likeness of the used robots have not been verified empirically as there are no reported methods so far, that allow to determine the perceived degree of human-likeness evoked by an artificial or natural entity.

At the beginning of each interaction, participants were informed about their upcoming game partner by a picture, displayed on their notebook screen. After 2000 ms the picture disappeared and a central crosshair on the computer screen indicated the start of a series and prompted the participants to find their decision (either pressing the left or right keyboard button; see above). The central crosshair disappeared after 1500 ms and was followed by an accumulated pay-off feedback for the current series (1000 ms). The accumulated pay-off feedback enabled participants to draw exact inferences about the partner's (i.e. CP, FR, AR or HP, respectively) latest response selection. The participant's pay-off was indicated by the lower numbers, the game partner's pay-off by the upper numbers. During the low level baseline no numeral response feedback was given. Instead two crosses replaced the numbers on the upper and lower side of the bar.

The briefing pursued two goals: firstly, familiarizing participants with the decision matrix and secondly, triggering a strong attachment to their game partners.

### fMRI setting

After the briefing the participant passed on to the MR-scanner located in the adjacent room. The experimenter gave last instructions and clarified that the participant understood the winning matrix as well as the converse demand to both “win a series and reach a virtual highscore”. Confederate, robots and the computer partner “remained seated” outside the scanner room. The mocked connecting cable was reset and “connected” to the MR presentation computer in the presence of the participant to make them believe that they would really and directly interact with all four game partners similar to the prior briefing session.

With the beginning of the functional imaging recording a randomized script file (the experiment was performed using Presentation® software; Version 0.70, www.neuro-bs.com) was started with the projection of a condensed summary of the instructions onto the MR-compatible video goggles (Resonance Technology). The timing and setting of the fMRI session were the same as during the briefing. Participants indicated their decision (cooperation or defection) by pressing one of two buttons with their right index finger on a fiberoptic custom-made response box. The behavioural outcomes of each single game were recorded. A series of nine games (i.e. one game equals one response) per condition (i.e. CP, FR, AR, HP or control) completed one block. Overall, participants played ten blocks per condition. After scanning participants were asked to fill out a questionnaire about their impressions of the task and game partners. In a debriefing participants were further interviewed about their impressions about the “strategy use” of their putative game partners as well as about their pleasure interacting with each game partner. Finally, participants were informed about the mocked scenario and the idea behind.

### Image Acquisition

All scans were performed on a 3T whole body scanner (Phillips Medical Systems, Achieva, Best, Netherlands) using standard gradients and a standard quadrature head coil located at the University Hospital Aachen. Subjects lay in the supine position, while head movement was limited by foam padding within the head coil. In order to ensure optimal visual acuity participants were offered fMRI-compatible glasses that could be fixed to the video goggles. For each subject, we acquired one series of 870 EPI-scans. Stimuli were presented in a blocked design fashion, with ten blocks per condition (CP, FR, AR, HP and control) and a block length of nine single games (one game lasting 2500 ms).

Functional scans covered the whole brain, including five initial dummy scans parallel to the AC/PC line with the following parameters: NS = 32; ST = 3.5 mm; IG = 3.75 mm; MS = 64×64; FOV = 192×192 mm; TR = 2000 ms; TE = 30 ms; FA = 90°. For anatomical localization, we acquired high resolution images with a T1-weighted 3D FFE sequence (TR = 25 ms; TE = 4.59 ms; NS = 170 (sagital); ST = 2 mm; IG = 1 mm; FOV = 256×256 mm; voxel size = 1×1×2 mm). MR images were analyzed using Statistical Parametric Mapping (SPM5, ww.fil.ion.ucl.ac.uk) implemented in MATLAB 7.0 (Mathworks Inc., Sherborn, MA, USA).

### Image analysis

After discarding the first five volumes, all images were realigned to the first image to correct for head movement. Unwarping was used to correct for the interaction of susceptibility artifacts and head movement. Volumes were then normalized into standard stereotaxic anatomical MNI-space by using the transformation matrix calculated from the first EPI-scan of each subject and the EPI-template. Afterwards, the normalized data with a resliced voxel size of 4×4×4 mm was smoothed with an 8 mm FWHM isotropic Gaussian kernel to accommodate for inter-subject variation in brain anatomy. A general linear model (GLM) comprising four conditions (CP, FR, AR, HP) was specified for each participant. Based on the hypothesis of a linear increase of cortical activity with respect to the perceived degree of human-likeness we calculated a parametric modulation of functional images (i.e. CP modelled as 1, FR as 2, AR as 3 and finally HP as 4). An SPM5 group analysis was performed by entering these contrast images into random effects analyses using one-sample t-tests. The resulting group contrasts comprised CP>Control, FR<Control, AR<Control, HP<Control, the linear increase (CP<FR<AR<HP) and the parametric modulation. For all group analyses, we applied a voxel-wise threshold of *p*<0.05 (family wise error corrected) and a minimum cluster size of 10 coherent voxels.

Parameter estimates were extracted by moving the centre of ROI (ROI-radius = 10 mm) on the first-level (subject level) to the individual local maximum activation within the respective clusters of interest (i.e. TPJ, medFG, medPFG and Precuneus; see results section). Extracted betas then were averaged across subjects for each condition (CP, FR, AR and HP) vs. Control.

The reported voxel coordinates of activation peaks were transformed from MNI space to Talairach & Tournoux atlas space [54] by non-linear transformations (www.mrc-cbu.cam.ac.uk).

## Results

### Behavioral results

As a prerequisite to derive meaningful interpretations of the behavioral and functional imaging data on-line response behaviour and questionnaires indicated that all of the 20 participants believed in the setting, i.e. they believed to really interact with the partners on-line.

Neither reaction times nor button pressing behaviour differed significantly between conditions (reaction times: *F*(1,19) = .07; *p* = .98; button pressing behaviour: *F*(1,19) = .26; *p* = .85). Overall, participants played rather competitive with a ratio of around 60/40 (competitive/cooperative) decisions, irrespective of the partner being played.

In the debriefing questionnaire, fun, intelligence, competitiveness and sympathy towards the players was evaluated. Participants indicated having experienced linearly increasing fun in the interaction the more the respective partner exhibited human-like features, i.e. CP<FR<AR<HP (linear trend of perceived fun: *F*(1,19) = 19.06; *p*<.0001). Similarly, game partners were attributed increasing intelligence the more they appeared human-like (linear trend of attributed intelligence: *F*(1,19) = 9.21; *p*<.005). For both calculations, quadratic and cubic trends did not reach significance.

Regarding the perceived competitiveness of game partners, an ANOVA did not yield significant results. However, post-hoc paired samples t-tests revealed that BARTHOC Jr.'s response behaviour was witnessed as being more competitive than the computer's and functional robot's behaviour (BARTHOC Jr. vs. computer, paired samples t-test: *t* = 2.46; *p*<.05; BARTHOC Jr. vs. functional robot, paired samples t-test: *t* = 2.99; *p*<.01). Human opponent and BARTHOC Jr. were rated as equally competitive (BARTHOC Jr. vs. human partner, paired samples t-test: *t* = −1.00; *p* = .33). Relative to their own game behaviour, subjects perceived themselves as being similarly competitive as the human opponent as well as BARTHOC Jr., however the computer partner and the functional robot were regarded as significantly less competitive (self-assessment vs. computer partner and functional robot, respectively, paired samples t-tests: *t* = 2.27; *p*<.05; *t* = 2.67; *p*<.05).

Overall, there was a significant difference in the self-estimated sensation between winning (positively valued) and loosing series (negatively valued), irrespective of the actual game partner (paired samples t-tests: computer partner: *t* = 9.45; *p*<.0001; functional robot: *t* = 7.86; *p*<.0001; anthropomorphic robot: *t* = 8.03; *p*<.0001; human partner: *t* = 10.80; *p*<.0001).

Finally, human-likeness and sympathy were rated only for BARTHOC Jr. and the functional robot. Participants evaluated BARTHOC Jr.'s appearance as significantly more human-like as the functional robot (paired samples t-test: *t* = 9.28; *p*<.0001) and there was a trend towards appreciating BARTHOC Jr. as more sympathetic and friendly compared to the functional robot (paired samples t-test: *t* = 1.88; *p*<.10).

Importantly, all of these evaluations must be ascribed to the different appearance of game partners as their response behaviour was, unknowingly to the participants, purely random (see methods section). Interestingly, some participants directly anthropomorphized BARTHOC Jr. during the briefing by talking to him or insulting him (“this X always knows what I am playing”). After the fMRI scanning, participants spontaneously re-interacted with the human confederate (e.g. congratulated or provocatively teased him) and sometimes even with BARTHOC Jr. (e.g. by waving the hand). However, no such interactions occurred with respect to the functional robot or the computer partner.

### Neuroimaging results

#### Simple contrasts

**Figure 4:**
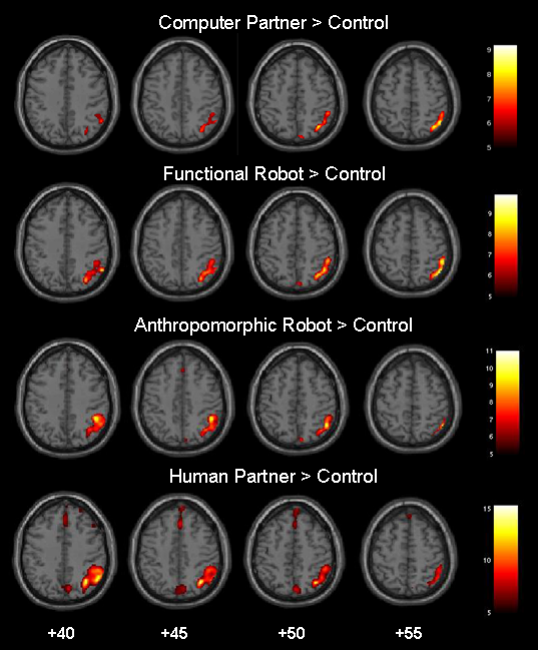
Regions activated more strongly during CP, FR, AR or HP as opposed to control. The activation map (*p*<0.05, family wise error corrected, minimal cluster size 10 voxels) is shown superimposed onto axial slices (z = +40∶5∶+55) of the T1-weighted SPM-template (http://imaging.mrc-cbu.cam.ac.uk/imaging/DisplaySlices).

**Figure 5:**
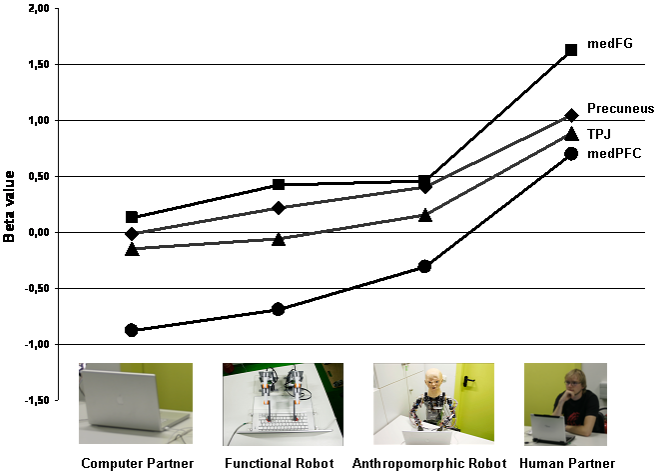
Averaged parameter estimates (beta values) for each condition derived from the individual local maxima activations (TPJ = temporo-parietal junction; medFG = medial frontal gyrus; medPFG = medial prefrontal gyrus; Precuneus).

Comparing each condition (CP, FR, AR and HP) with control yielded significant and concordant activation clusters located in the right temporo-parietal junction (TPJ) and the Precuneus (see Fig. 4; FWE corrected at *p*<.05). The cluster size of activation within the right temporo-parietal junction increased with human-likeness of the game partners. Similarly, activation of the medial prefrontal gyrus (AR<Control; HP<Control) extending into the medial frontal gyrus (FR<Control; AR<Control; HP<Control) and bilateral dorso-lateral prefrontal cortex (DLPFC) (FR<Control; AR<Control; HP<Control) manifested only for the more human-like game partners (see Fig. 4; Table 1). The human condition yielded strongest and most extended activation spreads, including all cortical regions detected within the three other comparisons. Regarding parameter estimates within all four regions (i.e. TPJ, medFG, medPFG and Precuneus) the results exhibit a significant linear trend corresponding to the perceived degree of human-likeness of game partners (linear trend of beta values for TPJ: *F*(1,76) = 20,85; *p*<.0001; linear trend of beta values for medFG: *F*(1,76) = 18,30; *p*<.0001; linear trend of beta values for medPFG: *F*(1,76) = 26,59; *p*<.0001; linear trend of beta values for Precuneus: *F*(1,76) = 18,67; *p*<.0001; see Fig. 5). Quadratic and cubic trends did not reach significance.

**Table 1:** Activation peaks with their localization.

	BA	Coordinates			t-value	No. voxels
		x	y	Z		
**Computer Partner>Control**						
Right Superior Parietal Lobule	40	36	−60	51	5.61	664
		46	−52	52	5.55	
		55	−52	41	5.09	
Precuneus	7	8	−73	52	4.86	21
**Functional Robot>Control**						
Right Inferior Parietal Lobule	40	46	−54	52	5.83	1123
		50	−40	52	5.69	
		55	−54	41	5.67	
Right Middle Frontal Gyrus	46/10	44	48	22	5.19	55
Left Inferior/Middle Frontal Gyrus	10	−40	50	−3	5.11	43
Precuneus	7	8	−73	52	4.72	29
**Anthropomorphic Robot>Control**						
Right Inferior Parietal Lobule	40	48	−52	50	6.10	1206
		46	−43	41	5.91	
		53	−51	38	5.37	
Precuneus	7	8	−75	50	7.19	39
Medial Frontal Gyrus	8	2	33	39	6.80	32
Left Middle Frontal Gyrus	10	−38	52	−1	6.62	17
**Human Partner>Control**						
Right Inferior Parietal Lobule	40	36	−58	43	6.95	2602
		53	−52	39	6.64	
		53	−41	35	6.25	
Right Superior Frontal Gyrus	40	38	50	20	5.57	856
Right Middle Frontal Gyrus	10	34	57	10	5.15	
		24	59	19	4.90	
Medial Frontal Gyrus/Anterior Cingulate Gyrus	6/9/32	2	38	29	5.53	586
Medial Frontal Gyrus	8	4	31	41	5.34	
Medial Frontal Gyrus	8/9	8	55	41	5.22	
Left Superior Frontal Gyrus	11	−36	46	−14	5.50	11
Precuneus	7	6	−71	51	5.41	372
		10	−68	42	5.26	
Left Middle Frontal Gyrus	10	−34	58	3	5.28	87
		−34	49	5	4.57	
Superior Frontal Gyrus	6	4	30	57	5.18	48
Left Inferior Frontal Gyrus	13/47	−34	21	−11	5.14	84
Right Superior Temporal Gyrus	38/47	53	19	−13	4.98	174
Right Superior Frontal Gyrus	6/8	20	22	58	4.91	85
Right Middle Frontal Gyrus	6/8	28	18	54	4.75	
Left Middle Frontal Gyrus	46	−46	42	18	4.56	14
**Humanlikeness (CP<FR<AR<HP)**						
Right Temporo-Parietal Junction	39	55	−53	21	6.83	313
Right Angular Gyrus	40	51	−66	35	5.78	
Right Superior Medial Frontal Gyrus	8	8	45	49	4.49	29

Significance level and the size of the respective activation cluster (number of voxels) for CP, FR, AR and HP as opposed to control (p<0.05, family wise error corrected, minimal cluster size 10 voxels) and parametric modulation of human-likeness, i.e. CP<FR<AR<HP (p<0.05, family wise error corrected, minimal cluster size 10 voxels). Coordinates are listed in Talairach and Tournoux (1988) atlas space. BA is the Brodmann area nearest to the coordinate and should be considered approximate.

### Parametric modulation

**Figure 6:**
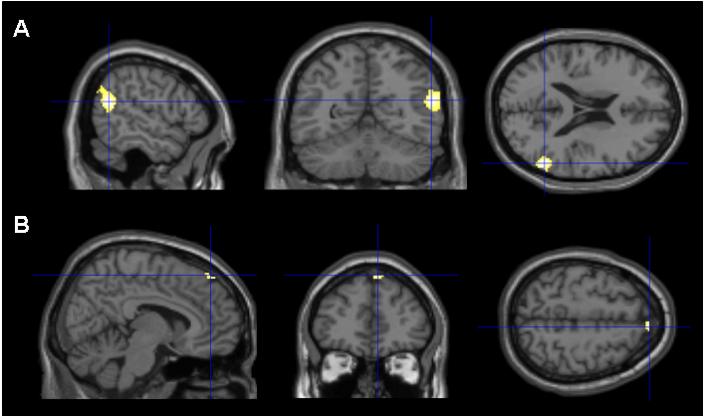
Parametric modulation of cortical activity corresponding to the hypothesized increase of ToM activity in line with game partners displaying increasing degrees of human-likeness (CP<FR<AR<HP). The crosshair is located at the local maxima activations in A: the right temporo-parietal junction (TPJ) and B: the superior medial frontal gyrus. The activation map (*p*<0.05, family wise error corrected, minimal cluster size 10 voxels) is shown superimposed onto three dimensional slices of the T1-weighted SPM-template.

Activity related to the parametric modulation (modelled as CP = 1; FR = 2; AR = 3; HP = 4) yielded highly significant activation clusters in two regions: the right temporo-parietal junction [BA 39/40, coordinates: x = 55, y = −53, z = 21; t = 6.83] and the medial frontal gyrus [BA 8, coordinates: x = 8, y = 45, z = 49; t = 4.49], both of which constitute the classic ToM network (see Table 1; Fig. 6). An opposite modelling, termed “counter-anthropomorphism” (i.e. (modelled parametrically as CP = 4; FR = 3; AR = 2; HP = 1), did not yield any significant activation, even by applying a more liberal t-threshold.

## Discussion

The objective of the present study was to investigate human-machine interaction and the impact of human-likeness on mental state attribution to *someone* or *something*. Especially in view of the rise of human-robot interactions in near future (e.g. application as caretaker, nurse, soldier, sex worker etc.), the present study sheds light onto the question of how humans perceive robotic machines. In the introduction we questioned whether humans think that “machines can think”? Would humans ascribe futuristic machines, as e.g. social, humanoid robots mental qualities? These questions are also important with respect to robotic design. One important question in the design of dialog modelling is how to communicate the internal system state of a robot in a way that is understandable to the human user? In order to successfully interact with a robot a human user must have a rough understanding of how the robot “thinks”. Thus, do human subjects attribute similar reasoning processes to a robot as they would to another human being? If so, what factors play a role in this attribution? The answers to these questions will heavily influence the way robots' physical and “mental” behaviour are designed in the future.

With a mocked highly interactive game scenario confronting human participants with four interaction partners – a computer, a functionally designed robot, an anthropomorphic robot and a human confederate – we could demonstrate that participants increasingly engaged cortical regions corresponding to the classical Theory-of-Mind network the more the respective game partners exhibited human-like features.

Thus, the same cortical network contributing to mental state attribution in implicit human-human interactions, i.e. right posterior superior temporal sulcus (pSTS) at the temporo-parietal junction (TPJ) and the medial prefrontal cortex (mPFC), was activated in the human-machine interactions. These findings were highly robust and could not be explained by behavioural or strategic alliances during the game as the response behaviour of all putative opponents was, unknowingly to the participants, randomized in advance. Thus pure intentional stance could be assessed, i.e. the disposition to treat an entity as a rational agent, possessing particular beliefs, desires and intentions [22], [55]. Further, as the game scenario of the current study equalled classical iterated Prisoner Dilemma Game (PDG) matrices, mentalizing performance was measured implicitly thereby circumventing socially desirable behaviour [18]–[20], [24], [25].

Regarding the participants' behaviour, the way participants interacted and treated all four game partners totally supported the high ecological validity of the study design. Notably, none of the 20 participants indicated having seen through the mocked setting. Quite the contrary, participants applied similar strategies for all four opponents as documented by the same ratio of competitive/cooperative decisions, while the time of response selection did not differ between interaction partners. Further, behavioural indices clearly documented the hypothesized direction of the effect of human-likeness: the more a game partner exhibited human-like features (CP<FR<AR<HP) the more the participants indicated having enjoyed the interaction and the more they attributed intelligence to their opponent.

Compared directly, participants rated the anthropomorphic robot as being more competitive and less cooperative as opposed to the functional robot and the computer. Interestingly, the putative human partner and the anthropomorphic robot were not perceived differently with respect to competitiveness. Further, a trend revealed the anthropomorphic relative to the functional robot as being more sympathetic and pleasant to interact with.

Accordingly, the appearance of the anthropomorphic robot was valued as more human-like and wins against him evoked more positive feelings compared to winning against the functional robot or the computer. These findings are fascinating with respect to the fact that all game partners solely played random sequences: hence, any differences in evaluations must be attributed to their differing appearance.

To be able to interpret and place the results of the current fMRI study into a broader context, the results of research documenting similar ToM effects based solely on computer or human opponents had been replicated in our setting [19], [20], [24]. Contrasting each of the four experimental conditions with control, basic ToM network activity (i. e. right temporo-parietal junction) could be detected for each comparison. However, only the anthropomorphic robot BARTHOC Jr. and the human partner elicited additional medial prefrontal gyrus activation at a more conservative statistical threshold. However, lowering the applied threshold yielded to medial prefrontal gyrus activations in the computer and functional robot condition as well, thus validating recent findings on human-computer interactions [19], [20], [24]. Another reason for the somehow smaller activation cluster in the medial frontal cortex could be the nature of the iterated PDG. Previous studies documented medPFG activity to be rather associated with competitive than cooperative behaviour [21]. The game matrix and instruction of the current study, however, was fitted to yield a levelled out playing. With a cooperation rate of 40% (compared to rates of 10–20% in other studies) the lower recruitment of the medial prefrontal cortex might be explained. The Precuneus activation detected in all comparisons is supposed to relate to mental imagery, self-processing operations and experience of agency [13], [56], [57].

Regarding the parametric modulation of the functional imaging data the perceived increase of human-likeness of game partners correlated significantly with only two cortical regions: the right temporo-parietal junction (TPJ) and the medial prefrontal cortex (medPFC), both areas constitute the classical mentalizing network. The TPJ contributes to processing naturalistic biological motion, human eye gaze observation [31] and plays a significant role in distinguishing self-/other-produced actions, termed agency [32]–[35]. According to Decety and co-workers the TPJ in conjunction with medPFC is activated when we distinguish the perspectives of the self from others, a finding perfectly displayed by the results of the present study [58]. Hence, we speculate that participants increasingly attributed agency to their game partners the more they exhibited human-like features. Most prominently agency is attributed to humans, but also inanimate objects [59] or even moving shapes [17] are regarded as exhibiting self-initiated actions [12]. Furthermore, mental simulations of their respective game partners actions (i.e. button pressing behaviour as observed during the briefing) likely contributed to the observed strong TPJ activity [60].

With respect to the linear increase of medial prefrontal cortex (medPFC) activation, we propose that “switching into the world” of the respective game partner succeeded easier the more human-like the game partner was perceived and therefore yielded stronger activations. Thus, anticipating what an agent is going to do next by considering what he or she would do being in the same situation also accounts for non-human partners [12], [36]. Rilling and colleagues chose the same line of reasoning for their human-computer interactions suggesting that either these brain regions contribute to reasoning about unobservable states of nonhuman systems, or that participants imbued their computer partners with human attributes (Rilling 2004).

As the medial prefrontal cortex activation detected in the present study was located at the dorsal and most posterior portion of the prefrontal cortex we propose that this region functions as an integration site bridging mental state attribution of similar/familiar to dissimilar/unknown others, thus human-likeness. This position is supported by recent studies reporting activity increases of the most inferior region of the medPFC during perspective taking of similar others [59], while dissimilar and unknown others rather activated the dorsal and most posterior region of the medPFC [12], [27].

### Implications and Conclusion of the study

To summarize the present study provides first evidence that the degree of human-likeness of a counterpart modulates its perception, influences the communication and behaviour, biases “mental” state attribution and, finally, affects cortical activity during such interactions. Here we show that this modulation is linear, thus the more a vis-a-vis agent or entity exhibits human-like features, the more we build a model of its “mind”. This process occurs irrespective of its behavioural responses and independently of whether we interact with real human partners or “just” machines. Returning to the question raised in the introduction, we would argue that humans implicitly ascribe automated machines, such as e.g. humanoid robots mental states, though on a lower level. However, as behaviour (embodiment) and appearance (anthropomorphism) modulate the degree of human-likeness one could speculate that with the rise of robotic development perfectly humanoid robots are being constructed (as e.g. Repliee Q1Expo). The findings also allow the conclusion that humans are able to perceive how “human-like” a robot is and that a less human-like looking robot might thus follow processing strategies that are alien to the human. This finding would thus suggest that the exterior of a robot should mirror its interior in that a more human-like looking robot should exhibit also more human-like behaviour.

To note, the present data do not provide any hints which particular morphological feature of the (robotic) interactors is crucial to ascribe a ToM. Future studies will need to address this issue in a more detailed way. However, as we hypothesised above, the degree of human-likeness might determine ToM.

Thus, we expect the results of our study to have an impact on long-lasting psychological and philosophical debates regarding human-machine interactions. Moreover, the findings of the present study will affect the designing of robots that are utilized for direct human-robot interactions. Finally, as psychiatric patients with autism spectrum disorder (ASD) have interestingly been found to prefer robotic to human partners in an initial interaction, though exhibit profound impairments in general mentalizing ability [25], it is possible that this patient group might have facilitated mentalizing when engaging with robotic relative to human interactors. Testing this hypothesis will have great importance for the clinical application of humanoid robots in ASD therapy.

As human-robot communication will play a key role in future (as prophesized in media reports, e.g. “the robot revolution is upon us” [61]), the present study may help to integrate theory and research from neuroscience and social robotics in order to place this work in a broader conceptual framework and promote synergy across fields.

## Supporting Information

Video S1 Setting of the briefing.
(3.37 MB MOV)
